# An Unusual High Bifurcation of the Brachial Artery: A Cadaveric Case Report

**DOI:** 10.7759/cureus.55681

**Published:** 2024-03-06

**Authors:** Kanchan Jangir, Brij Raj Singh, Nancy Nair, Ankit Badge

**Affiliations:** 1 Anatomy, Datta Meghe Institute of Higher Education and Research, Nagpur, IND; 2 Anatomy, Datta Meghe Institute of Medical Sciences, Nagpur, IND; 3 Clinical Embryology, Datta Meghe Institute of Higher Education and Research, Nagpur, IND; 4 Microbiology, Datta Meghe Institute of Higher Education and Research, Nagpur, IND

**Keywords:** clinical implications, cadaveric dissection, anatomical variation, brachial artery, high bifurcation

## Abstract

The human vascular system exhibits a remarkable degree of anatomical variability, with deviations from conventional arterial branching patterns occasionally encountered. Among these variations, the atypical bifurcation has drawn attention for its infrequent occurrence and potential clinical implications. This study investigates the rare anatomical variation of high bifurcation seen during cadaver dissection in the brachial artery. It emphasizes the relevance of understanding such variations in established vascular anatomy and their clinical implications. Detailed findings from the dissection of the upper limbs, which reveal a high bifurcation in a 40-year-old male cadaver, are presented. The report highlights unique anatomical variations, including a superficial path. The conclusion underscores the rarity of this high bifurcation and its potential impact on medical procedures. It stresses the importance of healthcare professionals being aware of and prepared for such anatomical variations for optimal patient care. In order to manage potential difficulties during medical operations affecting the circulatory system and eventually enhance patient outcomes, it is necessary to understand these deviations.

## Introduction

The human body's vascular system consists of a vast network of vessels essential to the circulation system's ability to transport blood. Arteries, veins, and capillaries collectively play an important role in the balance of biochemical activity, growth and development, absorption of nutrients such as vitamins and minerals, elimination of waste products, and cell homeostasis [1]. The human vascular system exhibits a remarkable degree of anatomical variability, with deviations from conventional arterial branching patterns occasionally encountered. Among these variations, the atypical bifurcation has drawn attention for its infrequent occurrence and potential clinical implications [2]. There are two possible outcomes for the brachial artery (BA): it can be absent or split into a higher and lower level, as well as a BA [3]. This case report aims to illuminate a specific instance of this arterial variation - a BA identified during cadaveric dissection.

At the bottom edge of the teres major muscle, the axillary artery (AA) extends into the BA. It descends and moves laterally to the front of the brachium, passing over the elbow joint. At the level of the radius neck, it divides into two terminal branches, the ulnar and radial arteries. This study shows a rare deviation in which the bifurcation point occurs at a level higher than expected and emphasizes the importance of a comprehensive knowledge of vascular anatomy.

An in-depth understanding of normal anatomical variation is necessary to perform various surgical treatment procedures and other medical therapies [4]. This case report will provide significant knowledge about the vascular anatomy of the upper extremities.

## Case presentation

During a routine dissection of the upper limb in the anatomy dissection lab for MBBS I-year students at Datta Meghe Medical College, Nagpur, an unusual arterial variation was observed in a 45-year-old male cadaver preserved in formaldehyde solution.

Dissection was started from the anterior compartment of the brachium with a skin incision with the help of forceps and a scalpel. In the superficial fascia, we demonstrated the median cubital vein, which is used for IV injections. Then the deep fascia and biceps brachii (BB) muscles were removed laterally to observe the BA, which was covered with bicipital aponeurosis. The AA and vein are closed within the axillary sheath. Dissection of the axilla and its contents will be facilitated by leaving the brachial plexus and AA intact while the axillary veins and their tributaries are removed. The AA was surrounded by the brachial plexus and, during the dissection of the AA, the brachial plexus.

**Figure 1:**
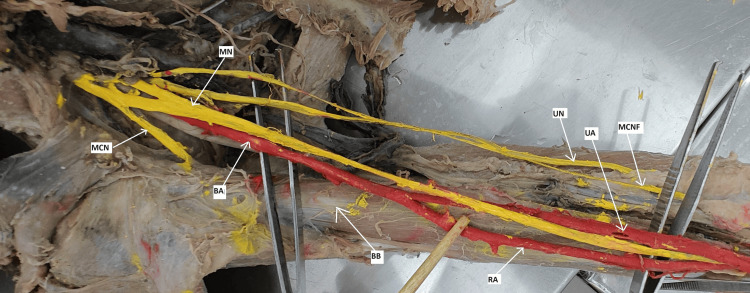
HBBA in a cadaver Red color shows the arteries and yellow shows the nerves MN: median nerve, MCN: musculocutaneous nerve, BA: brachial artery, UA: ulnar artery, RA: radial artery, UN: ulnar nerve, MCNF: median courteous nerve of forearm, BB: biceps brachii, HBBA: high bifurcation of the brachial artery

During dissection of the anterior compartment of the brachium of the right UL, the AA descends into the principal BA. Anteriorly, in the middle of the arm, it is crossed by the median nerve (MN) from the lateral to the medial side. Medially, in the upper part, it is related to the ulnar nerve (UN), and in the lower part, it is related to the MN. Laterally, it is related to the musculocutaneous nerve (MCN) and BB muscles, as shown in Figure 1. The BA bifurcation is 7.5 cm from the lower border of the teres major muscle and forms the radial and ulnar arteries. The radial artery allowed a course along the superficial surface of the brachialis muscle in the arm, traversed the cubital fossa, and then entered the deep surface of the brachioradialis muscle with the radial nerve in the antebrachium. The ulnar artery runs in the cubital fossa and travels deep to the deep head of the pronator teres; it is the continuation of BA in the antebrachium. There were no aberrant vascular observations in the left arm, and the radial and ulnar artery branching patterns in the antebrachium and hand were confirmed to be typical.

## Discussion

It is quite significant for surgical practice to recognize variations and paths of the UL arteries [3]. In addition, enhanced understanding in this domain improves diagnostic accuracy, mitigates risks associated with invasive procedures, and draws interest from anatomists and diverse medical specialists. These variations in anatomy are typically elucidated from an embryological perspective [4].

Clinical significance and variations

Understanding the typical branching pattern and course of the BA is crucial for interpreting clinical results and developing therapeutic strategies. There are several possible variations in the architecture of the BA, such as high bifurcations or abnormal branching patterns [5]. Awareness of such variations is crucial to avoiding complications during medical procedures. From a clinical perspective, the stethoscope is placed in front of the elbow joint, and the BA is crucial in monitoring blood pressure [6]. Oxygenated blood may flow through the elbow anastomosis that supplies the elbow joint. It reduces the possibility of inadequate vascularization by properly nourishing the ligaments and components of the joint capsule [7].

Supracondylar fractures of the humerus are commonly observed at a young age. Research has shown that vascular injuries occur in about 15% of pediatric patients with fully displaced supracondylar fractures, primarily affecting the BA. This injury can lead to vascular problems such as BA blockage and even limb amputation [8].

UL artery development

The intricate growth of the embryo results in a multitude of changes in the UL vascular system. Embryonic morphogenesis is a term used by clinicians who understand variations of the superficial BA (SBA). The AA originates from the seventh intersegmental (subclavian) artery and supplies each UL in the developing embryo. The AA grows distally, terminating in the palmar arch of the hand. Its main trunk forms the AA, the BA, the anterior interosseous artery, and the deep palmar arch. As a stable fetal channel, the SBA branches into the brachium superficial artery and the collateral branch before continuing as a portion of the RA and contributing to normal arterial morphogenesis [9].

Angiographic methods may be confused with high bifurcation of the brachial artery (HBBA). The unique location makes it difficult to identify and catheterize. The shallow path may result in extensive damage and unintentional intra-arterial injection [10]. However, this aberrant route facilitates cardiac catheterization and artery transplantation. Furthermore, the abnormal pattern of the upper arm artery, such as HBBA, may complicate surgical procedures such as distal biceps tendon replacement [11].

Prevalence of variations of HBBA

The prevalence of HBBA is relatively rare, estimated to occur in 1% to 5% of the population. Recognition of this variation is crucial for medical procedures. Various authors reported similar case reports to my finding (HBBA), which were reported by various authors. Wysiadecki et al. described the low origin of the radial artery and its embryology [12]. Pelin et al. reported that the high origin of the radial artery is 9.75% in an angiographic investigation and 14.27% in cadaver dissections [13]. Trifurcation of the BA occurs occasionally at 4.9 cm, as reported by Malci-Gürbuz et al. [14]. Gregory Tsoucalas et al. focused on the embryological aspect of the HBBA [9]. Chakravarthi et al. found an unusual bilateral accessory BA arising from the AA, and it is continuing in the forearm as a superficial accessory ulnar artery. They explain the morphology, embryology, and clinical implications of the BA [3].

## Conclusions

An unusual HBBA was observed in this cadaveric case report. This is a rare finding in cadavers. The unique branching pattern that was found might have an impact on the selection of incision sites or the location of grafts during revascularization surgeries, which could have an impact on surgical treatments involving the upper arm vasculature. To improve patient care and diagnostic procedures, more research on how often and the possible effects of such differences is needed.
